# Performing Simulated Basic Life Support without Seeing: Blind vs. Blindfolded People

**DOI:** 10.3390/ijerph182010724

**Published:** 2021-10-13

**Authors:** Santiago Martínez-Isasi, Cristina Jorge-Soto, Roberto Barcala-Furelos, Cristian Abelairas-Gómez, Aida Carballo-Fazanes, Felipe Fernández-Méndez, Candela Gómez-González, Vinay M. Nadkarni, Antonio Rodríguez-Núñez

**Affiliations:** 1 CLINURSID Research Group, University of Santiago de Compostela, 15782 Santiago de Compostela, Spain; smtzisasi@gmail.com (S.M.-I.); roberto.barcala.furelos@gmail.com (R.B.-F.); cristianabelairasgomez@gmail.com (C.A.-G.); aidacarballofaz@gmail.com (A.C.-F.); fernandez.mendez.felipe@gmail.com (F.F.-M.); candelagg723@gmail.com (C.G.-G.); antoniorodrigueznunez@sergas.es (A.R.-N.); 2 Faculty of Nursing, University of Santiago de Compostela, 15782 Santiago de Compostela, Spain; 3 Life Support and Simulation Research Group, Health Research Institute of Santiago (IDIS), 15706 Santiago de Compostela, Spain; 4 REMOSS Research Group, Faculty of Education and Sports Science, University of Vigo, 36005 Pontevedra, Spain; 5 University College of Nursing, University of Vigo, 36004 Pontevedra, Spain; 6 Department of Anesthesiology and Critical Care Medicine, Children’s Hospital of Philadelphia, University of Pennsylvania Perelman School of Medicine, Philadelphia, PA 3400, USA; nadkarni@email.chop.edu; 7 Department of Pediatrics, Children’s Hospital of Philadelphia, University of Pennsylvania Pereman School of Medicine, Philadelphia, PA 3400, USA; 8 Pediatric Intensive Care Unit, University Clinical Hospital of Santiago de Compostela, 15706 Santiago de Comopostela, Spain

**Keywords:** life support care, visually impaired persons, simulation training, cardiopulmonary resuscitation, heart arrest

## Abstract

Previous pilot experience has shown the ability of visually impaired and blind people (BP) to learn basic life support (BLS), but no studies have compared their abilities with blindfolded people (BFP) after participating in the same instructor-led, real-time feedback training. Twenty-nine BP and 30 BFP participated in this quasi-experimental trial. Training consisted of a 1 h theoretical and practical training session with an additional 30 min afterwards, led by nurses with prior experience in BLS training of various collectives. Quantitative quality of chest compressions (CC), AED use and BLS sequence were evaluated by means of a simulation scenario. BP’s median time to start CC was less than 35 s. Global and specific components of CC quality were similar between groups, except for compression rate (BFP: 123.4 + 15.2 vs. BP: 110.8 + 15.3 CC/min; *p* = 0.002). Mean compression depth was below the recommended target in both groups, and optimal CC depth was achieved by 27.6% of blind and 23.3% of blindfolded people (*p* = 0.288). Time to discharge was significantly longer in BFP than BP (86.0 + 24.9 vs. 66.0 + 27.0 s; *p* = 0.004). Thus, after an adapted and short training program, blind people were revealed to have abilities comparable to those of blindfolded people in learning and performing the BLS sequence and CC.

## 1. Introduction

Out-of-hospital cardiac arrest (OHCA) is a significant public health problem. In Europe, over 275,000 cases of OHCA are recorded every year with high mortality rates (around 75%) [1]. Mortality and outcomes are mainly associated with the time between the beginning of cardiac arrest (CA) and the start of cardiopulmonary resuscitation (CPR) [2]. Thus, to train laypeople in basic life support (BLS) skills in order to ensure an early response in case of OHCA, and consequently improve the victims’ survival and neurologic outcome, is a well-known statement supported by the scientific community [3,4,5]. Evidence indicates a clearly positive association between increased numbers of CPR by bystanders, access to an automatic external defibrillator (AED) and survival of OHCA [6,7]. Short training strategies have been suggested to be very effective, both for adults and schoolchildren inexperienced in life support [8]. However, despite their increasingly active presence as citizens, community CPR education has not focused on impaired people. There are very few references to CPR skills and/or training for groups with disabilities, such as people with Down syndrome or visual impairments, but the preliminary results of such studies are promising [9,10,11]. Therefore, it seems that even though scientific evidence supports CPR training for the entire population, the strategies implemented to date are not inclusive of all the groups that make up our society.

WHO reports that about 2 billion people worldwide suffer from vision impairment, of which 1 billion suffer from moderate or severe vision impairment or blindness [12]. People with visual impairments try to develop an active role in our society. Advocacy organizations for blind people work all over the world to improve their social inclusion and their access to the same education, social and leisure activities as people without visual impairments. In Spain, the non-governmental organization, the Spanish National Organization for the Blind (ONCE), performs a remarkable and multi-purpose function, with a history and range of projects worthy of note. As a reference, in 2017, ONCE had 72,097 members, from a range of ages, of which 20% were fully blind, and the remaining 80% had significant visual impairments [13]. Blind people (BP) are especially sensitive to helping others, and they claim their right to be considered as any other citizen. This includes learning to perform BLS and trying to initiate the recovery process of any CA victim.

Previous pilot and uncontrolled experience have shown the potential ability of blind or visually impaired people to learn the BLS sequence after participating in a general-purpose training activity, without specific adaptation to their condition [9]. There are currently no other references on CPR skills and/or training initiatives for the visually impaired. Therefore, with the hypothesis that a visual handicap is not a relevant barrier to perform standard-quality simulated BLS, the objective of this study was to compare the BLS skills of visually impaired people with a group of control blindfolded non-handicapped laypersons, after participating in the same adapted instructor-led, hands-on and real-time feedback training.

## 2. Materials and Methods

### 2.1. Design

This is a quasi-experimental trial with a convenience sample.

### 2.2. Participants and Selection Criteria

We invited to participate in the present study both blind or severely visually handicapped adults affiliated with the ONCE organization in Galicia (Spain), and adult people without visual or other handicaps, who were participating in vocational health training for laypeople at the high school Liceo la Paz, La Coruña, Galicia, (Spain). Blind people comprised the study group (BP), and non-handicapped people comprised the control group (BFP). They were informed about the study’s objectives and consented to be trained while their eyes were sealed with a blindfold (sleep mask).

The inclusion criteria for the BP group included having a severe visual impairment or total blindness, and being a member of ONCE. Both groups were required to be older than 18 years, and voluntarily accept participation in the study, signing the corresponding informed consent. The exclusion criteria for both groups were to have an associated psychical disability or a physical limitation for performing chest compressions (CC).

### 2.3. Intervention

Both groups received a 1 h theoretical and practical instructor-led training session in BLS, with real-time feedback. The BFP carried out the training with their eyes blindfolded during the whole session. The sessions were led by nurses with prior experience in BLS training of various collectives (health professionals, lifeguards, citizens, children and people with mental disabilities). The trainers also received specific pedagogical training focused on BP, provided by ONCE’s expert staff, and accordingly, they performed some modifications to their standard way of teaching. Briefly, these were: (a) direct supervision by expert teachers of BP; (b) student–teacher ratio of always less than 5 students per instructor, to facilitate student–teacher interaction and hands-on time; (c) real-time auditory feedback, encouraging tactile contact with the training materials, mannequin torso and an automated external defibrillator (AED) simulation device; (d) explanation of the different techniques and steps, considering the “blindness” of participants; and (e) with the AED, the same process of description and identification of the components was carried out to familiarize participants with the procedures, so that they could execute them correctly. 

Training sessions consisted of three steps. In the first, the participants identified the anatomical regions on their own body, together with an explanation of basic BLS sequence. In the second, they were encouraged to identify the anatomical regions on the training mannequin, and then performed all the actions to be taken in case of CA, including two minutes of continuous CC. Real-time verbal feedback quality was given by the instructor during the simulated BLS performance, and a metronome was used during CC training to improve rate compliance. In addition, to facilitate the learning and retention of this aspect, the popular song “La Macarena” was used as a mental memory aid [14]. In the third phase, the proper use of AED was taught and trained. An explanation of the components of the device was given, as well as the recommended sequence for its safe application. The instructor simulated on a dummy the actions to be followed in a hypothetical case of CA in which an AED might be available. After that, participants carried out the AED procedure, following the instructor’s verbal feedback. Finally, they were instructed to perform by themselves a complete BLS sequence, including 2 min of CC and subsequent use of the AED.

Thirty minutes after the training session, life support skills were evaluated during a simple simulation scenario, in which the BLS sequence of action was assessed: (a) securing the area; (b) assessing consciousness; (c) requesting the AED; (d) opening the airway; (e) assessing breathing; (f) calling the Emergency Medical Services; and (g) starting and maintaining CC for 2 min. 

### 2.4. Variables

We registered the sex, age, weight, height, body mass index (BMI) and previous BLS training of participants. The quality of CC was recorded quantitatively, using the Laerdal Resusci Anne mannequin with the PC/Wireless Skillreport version (12.0.0.2), configured according to the 2015 international BLS recommendations (depth: 50–60 mm; frequency: 100–120 compressions/min). A 45 kg compression spring, previously installed by the manufacturer, was used. The variables included were: global QCPR (%), time to start CC and time to discharge (TD) in seconds, CC time (%), CC with adequate hand position (%), mean CC depth (mm), CC with full chest recoil (%), correct CC by depth (%), correct CC by rate (%) and mean CC compression rate (CC/min).

### 2.5. Statistics

For the study of quantitative variables, the normal distribution was checked using the Kolmogorov–Smirnov or Shapiro–Wilk test. Quantitative variables were expressed by measures of central tendency and dispersion mean + standard deviation (SD). Qualitative variables were presented in terms of absolute and relative frequencies. Pearson’s chi-square statistic was used to study the association between categorical variables. The comparison of means was made using a T-test or Mann–Whitney test. Data processing and analysis were performed using the SPSS v.21.0 statistical package. A significance level of *p* < 0.05 was established.

### 2.6. Ethics

Participation was voluntary and no personal incentive for participation was given. All participants were informed about the aims and study protocol and provided written informed consent. The study respected the Helsinki Declaration and was approved by a local institutional review board (Research Ethics Committee of the University School of Nursing, University of Vigo, Vigo, Spain).

## 3. Results

The sample was composed of 59 subjects, 29 BP (16 male and 13 female, mean age: 53.7 + 12.3 years old) and 30 BFP (4 male and 26 female, mean age: 32.3 + 12.6 years old). Females were more frequent in the BFP group (86.7%) than in the BP group (44.8%) (*p* = 0.001). The BP were older (53.7 + 12.3 years old) than the BFP (32.3 + 12.6 years old) (*p* < 0.001). Weight was 79.5 + 12.5 kg for BP and 67.3 + 12.6 kg for BFP (*p* < 0.001). The groups had similar height (167.4 + 7.8 cm for BP and 163.2 + 7.8 cm for BFP).

**Figure 1:**
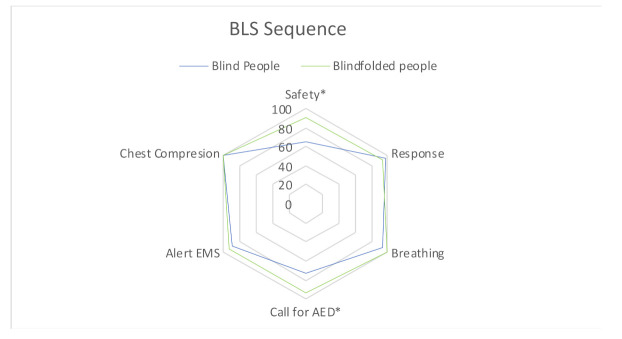
Comparison of BLS sequence performance between groups. * *p* < 0.005.

Regarding the BLS sequence (Figure 1), participants’ performed well, with a similar performance in both groups for response, breathing, EMS alert and CC, while we observed that BFP outperformed BP for “secure the scene” (19 (65.5%) BP vs. 27 (90%) BFP; *p* = 0.024) and “call for AED” (BP: 21 (72.4%) vs. BFP: 28 (93.3%); *p* = 0.035). The BLS sequence was performed without errors (fully following the correct order and performing all the procedures correctly) by 11 (37.9%) BP and 17 (51.7%) BFP (*p* = 0.119). Median time to start CC was 31 (range 26–41) seconds in BP and 33.5 (range 27–44) seconds in BFP (*p* = 0.844). 

**Figure 2:**
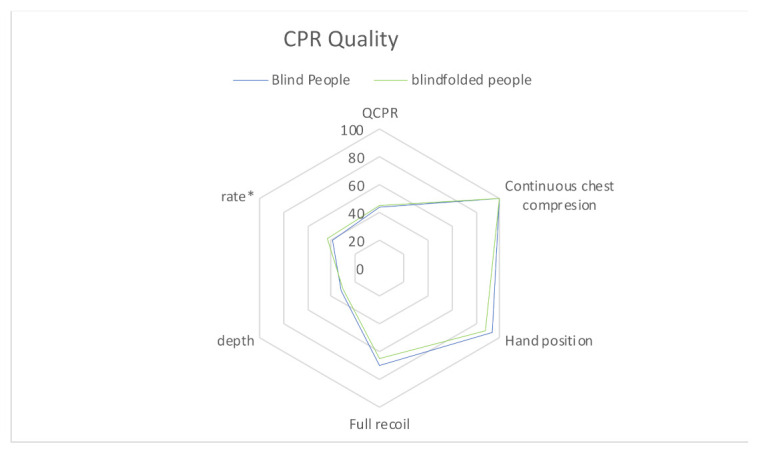
Assessment of chest compressions quality elements in blind and blindfolded people. * *p* < 0.005.

**Figure 3:**
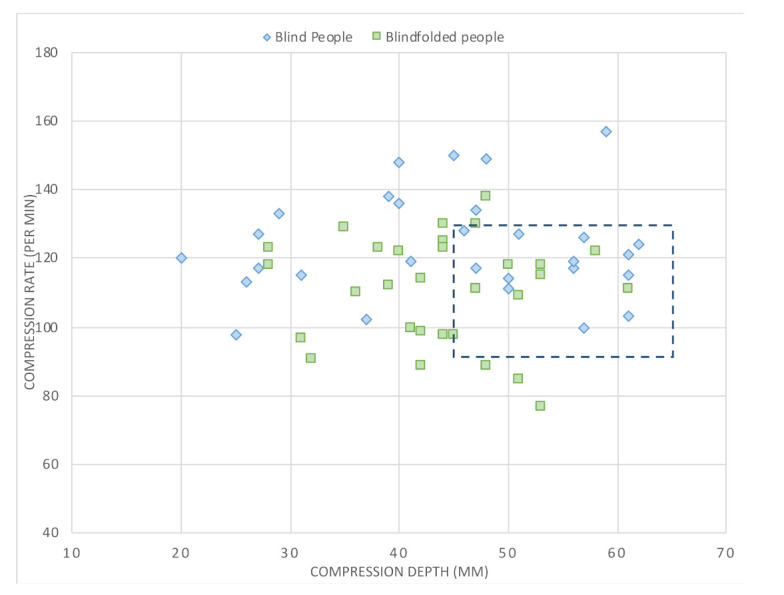
Scatter plot of the mean chest compression rate (compressions per minute) and depth (millimetres). The red square indicates the target, indicated by current guidelines. The dashed square represents an arbitrary expanded target with CC depth from 45 to 65 mm and CC rate from 90 to 130 CC/min.

Related to CC quality, the results are presented in Table 1 and Figure 2 and Figure 3. No significant differences between groups were observed for global QCPR, time to start CC, percentage of CC time, CC with adequate hand position, CC with full chest recoil and CC correct by depth and rate. Mean CC depth was also similar, but mean rate was significantly higher in BP than BFP (123 + 15.2 vs. 110 + 15.3 CC/min; *p* = 0.002). Time to discharge was significantly longer in BFP (86.0 + 24.9 vs. 66.0 + 27.0 s; *p* = 0.004). 

The percentage of BP subjects who achieved the recommended CC depth target was 27.6%, compared with 23.3% of BFP (*p* = 0.288). When the target was arbitrarily expanded to 10% below the lower limit and 8% above the upper limit (45–65 mm), it was achieved by 58.6% of BP and 43.3% of BFP individuals (*p* = 0.18) (Figure 3). For the CC rate target, it was achieved by 48.3% of BP and 36.7% of BFP participants (*p* = 0.025). When this target was arbitrarily expanded to 10% below the lower limit and 8% above the upper limit (90–130 CC/min), it was achieved by 72.4% of BP and 83.3% of BFP subjects (*p* = 0.24) (Figure 3).

## 4. Discussion

Our study, derived from a prior pilot BLS training experience with BP [9], is the only study published to date on the CPR skills of people with visual impairment. It shows that, despite significant visual impairment, laypersons are capable (after a simple, brief and adapted training programme) of performing the BLS sequence, including AED use at a similar quality to non-handicapped laypeople, who were requested to perform the sequence while blindfolded. Additionally, our data indicate that such a brief training session was not enough to learn how to deliver the strong CC needed to achieve the recommended CC depth target, a fact that has been reported in several studies, including in laypeople and even health staff [15,16,17]. To solve this limitation of training, new mannequins including real-time CC quality feedback features have been recommended, and are increasingly used in courses, both for laypeople and persons with a duty to assist [8,18]. 

Every citizen should be trained to perform BLS, regardless of their conditions and/or disabilities [9,10,11], not only because every person is a potential bystander first rescuer [6,7], but also as a way to promote the social inclusion and active participation of people with functional diversity. In this sense, our “proof of concept” study shows that by means of a simple adaptation of the methodology used in usual BLS training, it is possible to encourage people with visual impairment to acquire relevant BLS capabilities. Our teaching experience suggests that in the training of BP, it is very important that participants be in tactile contact with the training material at all times, as well as bearing in mind that BP learn differently and execute actions more slowly than a person without a visual disability. 

On the other hand, being blindfolded, non-visually handicapped people could improve their touching abilities, situation awareness, attention and communication skills, which hypothetically would focus their actions on the essential BLS steps and result in better learning [19,20]. 

Our results indicate that BP may have problems learning some steps of the BLS, namely “secure the scene”, something that seems quite logical when considering the importance of sight in being clearly aware of any situation. We consider that this is an unavoidable barrier for BP, who at this point must rely on the information received by other non-blind bystanders. On the other hand, the observed problems with the “call for AED” step in this group may not be clearly related to visual handicaps and could be attributed to a training deficit. In consequence, we consider that future training programs for BP should reinforce these topics, perhaps with specifically designed simulation scenarios. 

Regarding CC quality, our results indicate that BP are able to achieve a performance comparable to other laypeople, blindfolded or not [9,21,22,23,24,25] (Table 2). In fact, in our study, they outperformed in CC rates, both as a mean rate and as a percentage of CC, which were delivered at the recommended target rate. These results may be related to increased hearing perception and rhythm abilities, as well as paying more attention to the instructor feedback [26,27]. BP were also better than BFP at performing a quick AED discharge, a fact that could be explained by their prior abilities to pay attention and follow verbal and acoustic commands [28]. 

Our results indicate that for both BP and BFP, the chosen training program was insufficient to achieve the ability to perform CC with enough depth. Only around one quarter of subjects performed this specific skill according to current recommendations, and even after arbitrarily expanding the target by 10%, only around half of them succeeded. This training difficulty is well known (Table 2) and has been related to several factors, including subjects’ characteristics (age, BMI, fitness) and training methods (training times, feedback, etc.) that seem not to be related to blindness, and must be solved by means of specific reinforced training and re-training programs [8,29].

Our study has some limitations. This was a local experience including a limited number of subjects. The training methods were based more on expert opinion and teaching experience than on scientific evidence, which is lacking nowadays regardless. In consequence, it must be considered a proof-of-concept study that cannot be directly extrapolated to other settings and subjects. 

Our results suggest that further research is necessary in order to define the training time and potential impact of re-training sessions for laypersons, with and without visual impairment. As a future line of research, it would be interesting to analyze the usefulness of adapting the AED to the needs of the visually impaired.

## 5. Conclusions

After an adapted and short training programme, blind and blindfolded people demonstrated comparable abilities to learn and perform the BLS sequence and CC. The training method applied was insufficient to achieve the optimal CC depth, and both groups will need to re-train this specific skill. We believe that BP should be considered as candidates for BLS training like any other citizen, and we feel this activity would contribute to their social integration.

## Figures and Tables

**Table 1:** Chest compressions quality variables obtained by blind and blindfolded people. Results in mean (standard deviation).

Variables	Blind	Blindfolded	*p*
Global QCPR (%)	43.9 (38.1)	45.3 (31.1)	0.721
Time to start CC (seconds)	35.77 (12.6)	36.5 (14.4)	0.844
Time to discharge (seconds)	66.0 (27.0)	86.0 (24.9)	0.004
Compression time (%)	99,4 (1.2)	99.4 (1.4)	1
CC with adequate hand position (%)	93.1 (19.9)	88.6 (30.0)	0.626
Mean compression depth (mm)	44.7 (12.7)	43.8 (7.3)	0.761
CC with full chest recoil (%)	69.8 (36.6)	65.1 (36.5)	0.721
Correct CC by depth (%)	32.3 (38.0)	29.9 (35.7)	0.939
Correct CC by rate (%)	39.3 (38.0)	43.7 (37.0)	0.357
Mean compression rate (comp/min)	123.4 (15.2)	110.8 (15.3)	0.002

**Table 2:** Comparison of chest compressions quality standards obtained by BP, BFP and other laypersons after brief training.

Variables	BP *^,a^	BFP *^,a^	Teachers [15] ^a^	Cardiac Patients [16] ^b^	Participants Free Course [17]	Relatives [18] ^b^	Fisherman [19] ^a^	Nurse Students [20] ^a^
Global QCPR (%)	43.9 (38.1)	45.3 (31.1)	70.2 (31.1)	86 (71–92)		69 (20.5–89)	43 (10)	55.2 (24.9)
CC time (%)	99.4 (1.2)	99.4 (1.4)	98.8 (8.0)					74.7 (7.6)
CC with adequate hand positions (%)	93.1 (19.9)	88.6 (30.0)	97.7 (11.9)	100 (100–100)	99.3	100 (100–100)		98.2 (13.0)
Mean CC depth (mm)	44.7 (12.7)	43.8 (7.3)	48.21 (9.2)	56 (50–61)		46 (41–56)	56.5 (5)	44.2 (10.7)
CC with full chest recoil (%)	69.8 (36.6)	65.1 (36.5)	78.7 (29.7)	73 (25–98)	88.8	91 (45–99.5)	115 (16)	79.1 (26.9)
Correct CC by Depth (%)	32.3 (38.0)	29.9 (35.7)	46.9 (38.8)	39 (7–75)	75.7	38 (3–64.5)		32.6 (39.7)
Correct CC by rate (%)	39.3 (38.0)	43.7 (37.0)	64.2 (36.9)	55 (7–88)		18 (0.5–80.5)		50.4 (35.9)
Mean compression rate (comp/min)	123.4 (15.2)	110.8 (15.3)	109.7 (14.3)	106 (93–116)	92.5	106 (89–123)		113.1 (13.0)

* Current study, ^a^ mean (standard deviation), ^b^ median (IQR).
